# The Synthesis of Ru–Co–Oxalate MOFs for an Electrochemiluminescent Glyphosate Sensor

**DOI:** 10.3390/bios16030140

**Published:** 2026-02-28

**Authors:** Karina G. Espinosa-Cavazos, Joelis Rodríguez-Hernández, Carlos Gallardo-Vega, Carmen Alvarado-Canché, Marco Antonio Castillo, Roman Torres-Lubian, Perla E. García Casillas, Juan Carlos Anaya-Zavaleta, Antonio Ledezma-Pérez, Arxel de León

**Affiliations:** 1 Centro de Investigación en Química Aplicada, Boulevard Enrique Reyna 140, Saltillo Coahuila 25294, Mexico; karinaespinosa.c@gmail.com (K.G.E.-C.); joelis.rodriguez@ciqa.edu.mx (J.R.-H.); carlos.gallardo@ciqa.edu.mx (C.G.-V.); carmen.alvarado@ciqa.edu.mx (C.A.-C.); marco.castillo@ciqa.edu.mx (M.A.C.); roman.torres@ciqa.edu.mx (R.T.-L.); perla.garcia@ciqa.edu.mx (P.E.G.C.); 2 Micro and Nanotechnology Research Center, Universidad Veracruzana, Boca del Río 94294, Mexico; juancarlosanayazavaleta@gmail.com; 3 SECIHTI-Centro de Investigación en Química Aplicada, Boulevard Enrique Reyna 140, Saltillo Coahuila 25294, Mexico

**Keywords:** electrochemiluminescent, glyphosate, metal–organic frameworks

## Abstract

Cobalt–ruthenium bypiridine–oxalate metal–organic frameworks (MOFs) were synthesized via a solvothermal method with a custom-designed reactor that permits stirring, which can result in changes in the morphology of the structures. In this work, we performed a morphological and structural study of MOFs with varying tris(2,2,bipyridyl) and diclororuthenium(II) hexahydrate ${\left(Ru(bpy\right)}_{3}^{2+})$ concentrations, demonstrating changes in the size of the MOFs, and these MOFs were used as the luminescent materials in an electrochemiluminescent (ECL) system for glyphosate (Gly) detection, which acts as a coreactant in the light emission of ${Ru(bpy)}_{3}^{2+}$. Gly is the most commonly used herbicide worldwide, and our system has a calibration curve range of 10–70 ppm, with a detection limit of 7.6 ppm.

## 1. Introduction

Herbicides have emerged as powerful tools, enabling farmers to combat pests and weeds masterfully, thereby increasing crop production to new levels. However, significant concerns have been raised about the potential impact of herbicides on human health [1]. In recent decades, the widespread application of chemical herbicides has raised increasing concerns regarding their environmental and health implications. Among these substances, glyphosate has garnered particular attention not only for its extensive global use but also for its potential health risks. Owing to its broad-spectrum efficacy in weed management, glyphosate has become one of the most commonly employed herbicides in global agricultural practices [2]. It is effective in controlling diverse weed species, particularly in tea plantations. Nevertheless, its widespread and sustained use has attracted research and regulatory attention in various regions.

Glyphosate is generally considered a low-toxicity compound; however, due to its excessive use, it has accumulated to significant levels in the environment [3]. It is absorbed by soil, and its residues disperse through soil and inhibit the shikimic acid pathway, a metabolic process essential for many soil bacteria and fungi, similar to aromatic amino acid synthesis in plants. This inhibition decreases the activity of nitrogen-fixing and nitrifying bacteria, which disrupts the nitrogen cycle and diminishes soil fertility [4], leading to notable environmental impacts and harming human health. Some plants do not metabolize or excrete glyphosate and therefore accumulate it during their growth. Although glyphosate is not easily absorbed via the skin in humans, it is consumed indirectly through the consumption of crops that have been treated with it. The accumulation of glyphosate-based formulations found in urine and feces that do not degrade into other substances is a significant health hazard caused by glyphosate absorption or ingestion [5].

Some studies suggest that exposure to glyphosate is linked to various health risks—for example, the disruption of neurotransmission, DNA damage, and an increased risk of cancers, such as cutaneous melanoma and non-Hodgkin’s lymphoma; cancers of the breast, liver, and kidney; and urinary tract and thyroid cancer [6]. In addition, glyphosate has been connected to other health concerns, recently the effects of herbicide-based glyphosate on hepatic function were investigated by Qi et al., revealing the presence of oxidative stress and significant alterations that directly impair liver function in mice, thereby highlighting the potential risks associated with exposure to this herbicide [7].

Glyphosate is a potent polar compound with high hydrophilicity and lacks fluorophores and binding sites, which leads to difficulty in detecting this compound in complex environments. Conventional methods include thin-layer chromatography (TCL) [8], high-performance liquid chromatography (HPLC) [9], fluorescence [10], and nuclear magnetic resonance spectroscopy (NMR) [11], among others. These detection methods rely on complex, large-scale analytical instrumentation and high operational costs, which limit their applicability in high-throughput settings. This limitation is particularly critical in scenarios that require rapid and visually interpretable detection [12]. Therefore, alternative detection strategies for this herbicide have been actively investigated.

Metal–organic frameworks (MOFs) are materials that have crystal structures formed by the coordination of metal ions, including lanthanides or alkaline metals, with organic ligands such as carboxylates, phosphonates, and sulfonates [10,11]. Some of the components used in the synthesis of MOFs include metal ions with square, pyramidal, trigonal, bipyramidal, tetrahedral, and octahedral geometries, including Fe(III), Zr(VI), Cu(II), Mg(II), Ti(III), and Co(II), among others [12].

Currently, ruthenium-rich metal–organic frameworks stand out because of their unique chemical properties and are utilized in applications such as the development of dual-mode sensors [13,14]. Tris(2,2′-bipyridyl) ruthenium(II) ${Ru(bpy)}_{3}^{2+}$ is employed in electrochemiluminescence (ECL) because of its numerous advantages, such as chemical stability, small required doses, and superior biocompatibility in ECL analytical systems [15].

Additionally, metal–organic frameworks (MOFs) exhibit high specific surface areas, tunable porosities, and inherently diverse electronic and dimensional structures and have been widely applied in biosensing applications and drug delivery. ECL carriers can be incorporated into the framework or pores of MOFs [16,17,18].

To improve the ECL properties of MOFs, various researchers have focused on functionalization, particularly through the incorporation of inorganic molecular components. Coordination chemistry has proven to be a practical approach in this context. Notably, MOFs synthesized using oxalic acid have demonstrated the ability to encapsulate ${Ru(bpy)}_{3}^{2+}$ complexes for ECL sensing applications. This is attributed to the ability of oxalate ions to bridge metal centers via significant exchange interactions, especially with transition metals. The resulting coordination bonds enhance both the chemical stability and the ECL performance of the system [19].

**Scheme 1:**
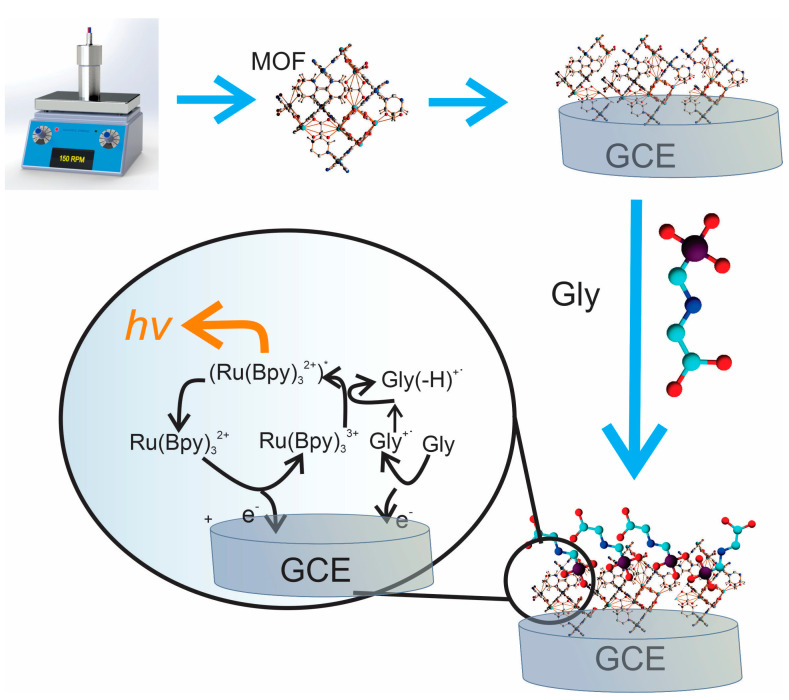
Illustration showing the reactor and ECL sensor for glyphosate detection. * symbolized a free radical.

In this paper, we report the synthesis of MOFs based on cobalt and ruthenium bipyridine complexes and oxalic acid. These MOFs were obtained with a novel custom-made reactor that combines resistance and a thermopar in the middle of the reactor. This reactor allows for stirring during the reaction. These MOFs were used as the luminescent components in an ECL sensor for glyphosate detection (Scheme 1). The results were used to construct a calibration curve in the range of 10–70 ppm, with an LOD of 7.6 ppm, achieving an improved assay performance.

## 2. Materials and Methods

### 2.1. Materials

Hydrochloric acid (HCl, 99%), N,N-dimethylformamide (DMF), cobalt nitrate (Co(NO_3_)_2_, 98%), oxalic acid ((COOH)_2_ 99%), tris(2,2,bipyridyl), diclororuthenium(II) hexahydrate ${Ru(bpy)}_{3}^{2+}$ (99.5%), and ethanol (CH_3_CH_2_OH, 99.99%) were acquired from Sigma-Aldrich Chemical Company (St. Louis, MO, USA; Burlingtom, MA, USA). Deionized water and carbon screen-printed electrodes, suministred by Dropsens, were from METRHOHM (Asturias, Spain).

### 2.2. Synthesis of Co(oxalate)-${Ru(bpy)}_{3}^{2+}$

The reaction was carried out with a modified version of a procedure from Wang et al. [20]. The MOF was synthesized as follows: 60 mg of oxalic acid, 9 mL of hydrochloric acid, 200 mg of Co(NO_3_)_2_, and ${Ru(bpy)}_{3}^{2+}$ in amounts of 30, 40, 60, and 80 mg in 40 mL of DMF and water (2:1, *v*/*v*) were combined in a custom-made design solvothermal reactor with hot resistance and a thermopar that permits magnetic stirring; the reaction was carried out at 333.15 K (60 °C) for 48 h. The product of this reaction was precipitated by centrifugation, washed with ethanol and deionized water twice, and then dried by lyophilization. The products of this reaction are referred to as Co-Ru-30, Co-Ru-40, Co-Ru-60, and Co-Ru-80, corresponding to 30, 40, 60, and 80 mg of the ruthenium complex, respectively.

### 2.3. Sensor Assembly

The sensor was assembled on a Dropsens DRP-110 screen-printed electrode, where the working and auxiliary electrodes were carbon and silver, as a reference electrode; on the electrode, 5 µL of dispersed solution in 0.1 M PBS (1 mg/mL) of MOFs Co-Ru-60 or Co-Ru-80 was added, and 5 µL of glyphosate solution dissolved in 0.1 mol L^−1^ PBS (pH 8) to different concentrations of 10, 20, 50 and 70 ppm was added. Finally, 10 µL of 0.1 mol L^−1^ PBS (pH 8) was added to the ECL measurement system. Cyclic voltammetry was performed within a potential window of 0 to 1.5 V at a scan rate of 0.02 V/S, which was realized with a µSTAT ECL from Dropsen METRHOHM, which combines ECL with a silicon photodiode and galvanostat/potentiostat.

### 2.4. Characterization Techniques

X-ray diffraction (XRD) was performed on an Eco D8 Advance instrument (Bruker, Billerica, MA, USA) in the range of 5–70 (2θ); the samples were ground with an agate mortar, and the loose powder was pressed into a diffractometer sample holder. X-ray powder diffraction patterns were collected using Bragg–Brentano geometry at room temperature with CuKα radiation (λ = 1.54183 Å) in an Ultima IV diffractometer. The absorbance spectra were recorded on a SHIMADZU UV-2401PC spectrophotometer (Shimadzu, Kyoto, Japan), the UV-Vis data were collected in the range of 250–1000 nm, and the emission spectra were acquired on a Horiba Duetta absorbance–fluorescence spectrophotometer (HORIBA Canada, Inc., Mississauga, ON, Canada) at an excitation wavelength of 450 to 500 nm at 10 nm increments. The FT-IR spectrum was collected on a Nicolet iS5 Thermo Scientific iD7ATR (Waltham, MA, USA) instrument in a range from 4000 to 600 cm^−1^, with 16 cm^−1^ resolution and 36 scans using germanium as the reference material. The morphology of the MOFs was analyzed via scanning electron microscopy on a FEI Quanta 200-3D instrument (FEI Company, Hillsboro, OR, USA). The size distributions were determined by statistical measurement of SEM pictures using free software ImageJ 1.53t (https://imagej.net/ij/).

## 3. Results and Discussion

**Figure 1:**
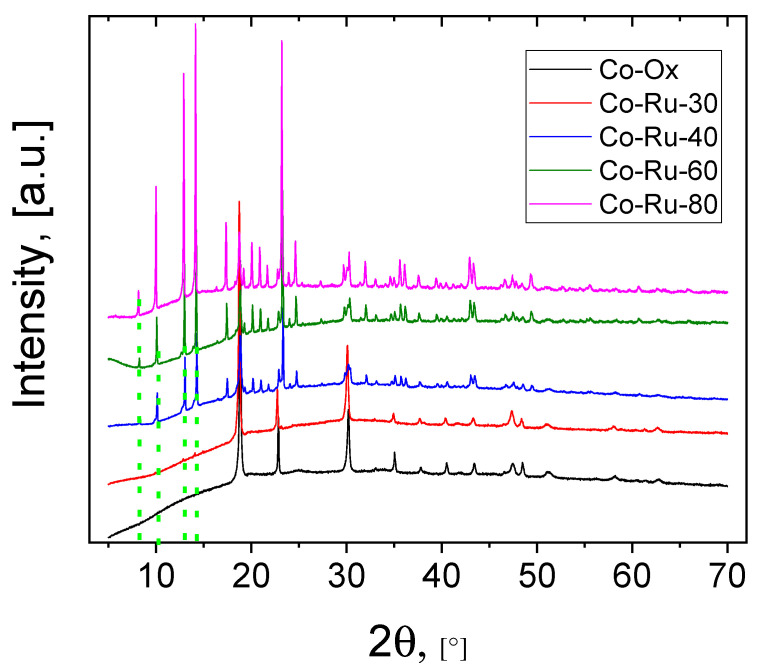
XRD patterns of Co-Ox, Co-Ru-30, Co-Ru-40, Co-Ru-60 and Co-Ru-80.

The patterns of the samples were recorded from 5 to 70° (2θ) in 0.02° steps and a 10 s counting time (Figure 1). The structural model to be refined was obtained from the crystallographic data reported in card number 936142 ([${Ru(bpy)}_{3}^{2+}$][Zn_2_(C_2_O_4_)_3_]) from the Crystal Structure Database (CCDC) from reference [21].

The results indicate that Co–oxalate has an oxalate structure, as per 004-0621-PDXL, indicating that the MOF has not yet begun to form. With an increasing ${Ru(bpy)}_{3}^{2+}$ concentration, reflections from the cubic structure begin to appear at 2θ = (8.4°), (10°), (12.8°), (17.4°), and (23.4°) for Ru-Co-30 and are more strongly defined at higher concentrations with greater reflections for Co-Ru-80. This finding corroborates MOF formation and indicates that ${Ru(bpy)}_{3}^{2+}$ plays a crucial role in MOF formation, as evidenced by the appearance of new reflections.

**Figure 2:**
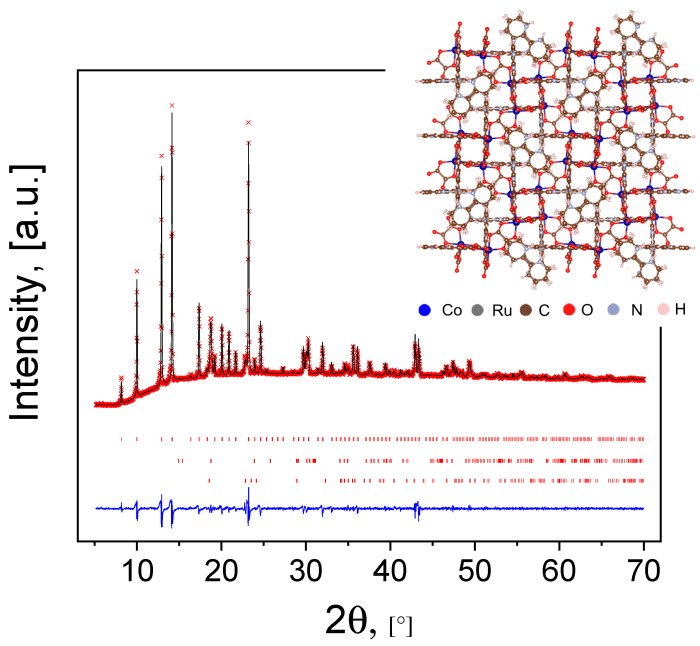
Experimental and fitted XRD powder patterns and their difference for Co-Ru-80. The inset shows the atom packing within the framework for the materials, experimental (black), calculated (red) and difference (blue).

The space group (P4_1_32) assignment was corroborated using the Le Bail method [22]. Structural refinement was carried out via the Rietveld method implemented in the FullProf program [23]. These materials are isostructural and crystallize in a cubic unit cell, in the P4132 space group, with cell parameters of a = 15.3302(4) Å (Ru-Co-60) and a = 15.3294(6) Å (Ru-Co-80). The XRD patterns (experimental, calculated and difference) for the compounds are shown in Figure 2.

**Figure 3:**
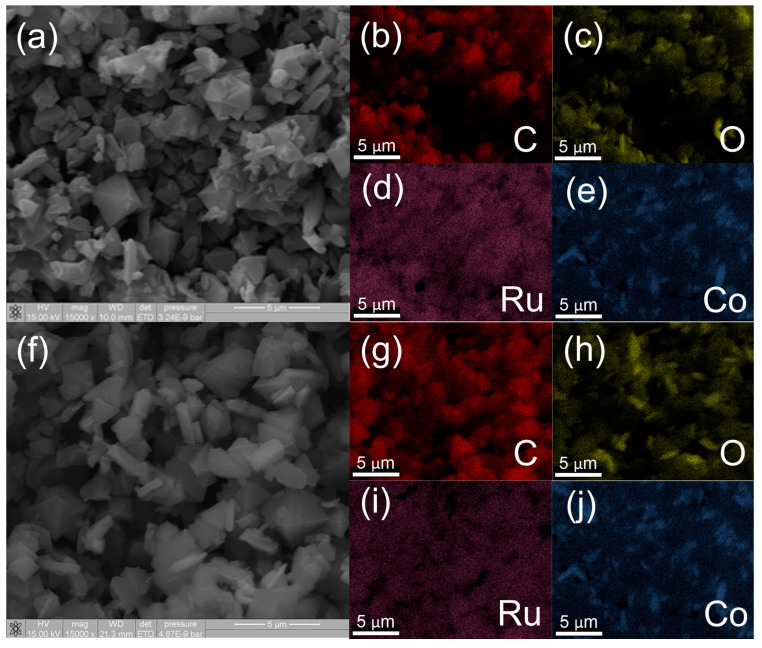
SEM-EDS micrographs and elemental mapping for Co-Ru-60, (**a**) complete image of the MOF, (**b**) identification of carbon, (**c**) corresponding oxygen present in the MOF, (**d**) the presence of ruthenium on the MOF, (**e**) the presence of cobalt on the MOF, (**f**) SEM-EDS mapping for Co-Ru-80 and image of the morphology of the MOF, (**g**) the presence of carbon in the MOF, (**h**) the presence of oxygen, (**i**) the presence of ruthenium and (**j**) the presence of cobalt in the MOF.

The Co–oxalate morphological analysis shows polymorphic MOF formation with a predominant octahedral structure at a ${Ru(bpy)}_{3}^{2+}$ concentration of 60 mg, resulting in an average size of 2.43 ± 0.61 µm (see Figure 3a). In contrast, the MOF formed with 80 mg of ruthenium does not show many octahedral structures, and the morphology changes to bar-like, indicating that ${Ru(bpy)}_{3}^{2+}$ changes the interaction between Co and oxalate, resulting in an average size of 2.23 ± 0.78 µm (see Figure 3f) [21]. When smaller amounts of Ru are used, the MOF size is larger than that at higher concentrations, measuring 4.20 ± 1.70 µm and 3.22 ± 1.18 µm at 30 mg and 40 mg of ruthenium, respectively (see Figure S1 and Table 1); the size of the structures was measured with free software Image J. This finding corroborates that the amount of ruthenium employed changes the interaction between cobalt and oxalic acid. The elemental mapping from the EDS analysis reveals the presence of Ru, Co, C, and O, with a uniform distribution for Co-Ru-60 and Co-Ru-80, where the Co-Ru-60 system was composed of carbon (33%), ruthenium (10%), oxygen (12%) and cobalt (40%), and the Co-Ru-80 system was composed of carbon (33%), ruthenium (15), oxygen (13%) and cobalt (31%) (see Figure S2), showing an increase in the Ru amount for Co-Ru-80. These results corroborate that the ruthenium complex interacts directly with Cobalt–Oxalate and confirm that the MOF can be used in electrochemiluminescence systems.

**Figure 4:**
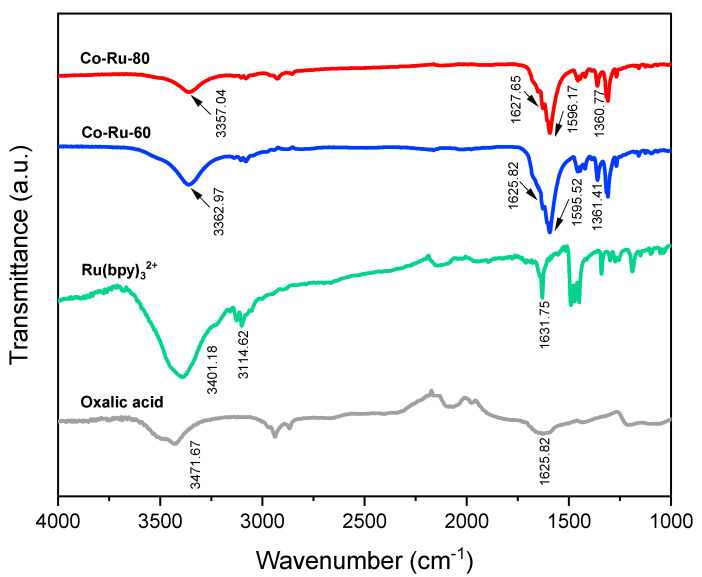
FT-IR spectra of oxalic acid, ${Ru(bpy)}_{3}^{2+}$, Co-Ru-60 and Co-Ru-80.

On the basis of the morphological and XRD analysis, it was concluded that the use of Co-Ru-60 and Co-Ru-80 resulted in better properties with small-sized materials. The Co-Ru-80 and Co-Ru-60 MOFs revealed similar peaks in the FTIR analyses (Figure 4). The peaks at 3362.97 cm^−1^ and 3357.04 cm^−1^ could result from the coordination upon forming a MOF architecture involving coordination interactions between cobalt–oxalate and ${Ru(bpy)}_{3}^{2+}$. This supports the proposed formation of an MOF architecture involving Co^2+^ ions, consistent with the FTIR spectra of oxalic acid and ${Ru(bpy)}_{3}^{2+}$ [14]. A noticeable shift is observed toward lower wavenumbers at 1595.52 cm^−1^ and 1596.17 cm^−1^, which are associated with the symmetric and asymmetric stretching vibrations of the C-O bond of the carboxylate group. The disappearance of the C=O asymmetric stretching vibration peak at 1625.82 cm^−1^ and 1627.65 cm^−1^ together with the symmetric stretching vibration peaks at 1361.41 cm^−1^ and 1360.77 cm^−1^, respectively, support the occurrence of the total deprotonation of carboxylic acid groups, enabling their attachment to metal centers [13,24].

**Figure 5:**
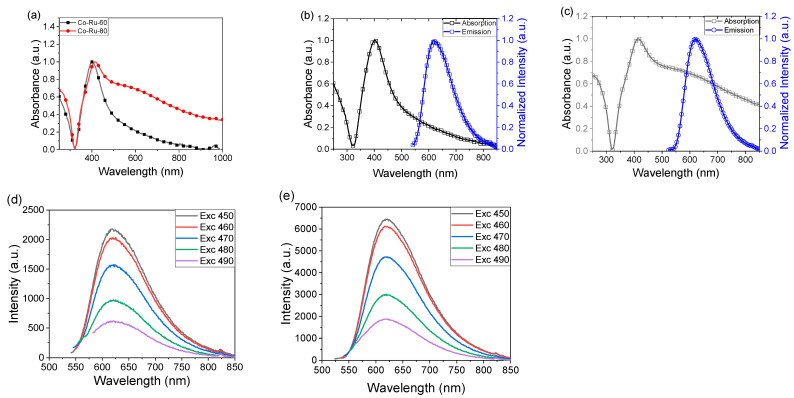
(**a**) UV-Vis absorption spectra for Co-Ru-60 and Co-Ru-80, (**b**) UV-Vis and emission spectra for Co-Ru-60, (**c**) UV-Vis and emission spectra for Co-Ru-80, and (**d**,**e**) excitation study at different wavelengths for Co-Ru-60 and Co-Ru-80 respectively.

The absorbance spectra for the Co-Ru MOFs are shown in Figure 5. These spectra show a transition at 400 nm for Co-Ru-60 and a bathochromic shift at 414 nm for Co-Ru-80 (Figure 5a). This transition corresponds to the metal-ligand complex, which does not appear as a shoulder between 505 and 1000 nm; this can be attributed to the MOF agglomerate structures that can overlap with the ruthenium transition. These results again corroborate the interaction between Co–oxalate and ruthenium. Figure 5b,c shows emission spectra that have a maximum at 621 nm for both systems; this emission is characteristic of ruthenium emissions, attributed to the triplet metal-to-ligand decay [25]. The peak at 621 nm remains constant for the systems and does not shift with increasing excitation, which corroborates the integrity of ${Ru(bpy)}_{3}^{2+}$ in the MOF, demonstrating no change in its emission properties (Figure 5d,e).

### Electrochemiluminescence Sensor Performance

The ECL performance for glyphosate detection was tested with the cobalt–oxalate MOF as the coreactant, where the ${Ru(bpy)}_{3}^{2+}$ complex present in the MOF is oxidized in the electrode to ${Ru(bpy)}_{3}^{3+}$, Gly is oxidized by ${Ru(bpy)}_{3}^{3+}$ and the Gly radical cation reduces the complex ${Ru(bpy)}_{3}^{3+}$, generating an excited state that produces light upon a return to the ground state [26,27].

**Figure 6:**
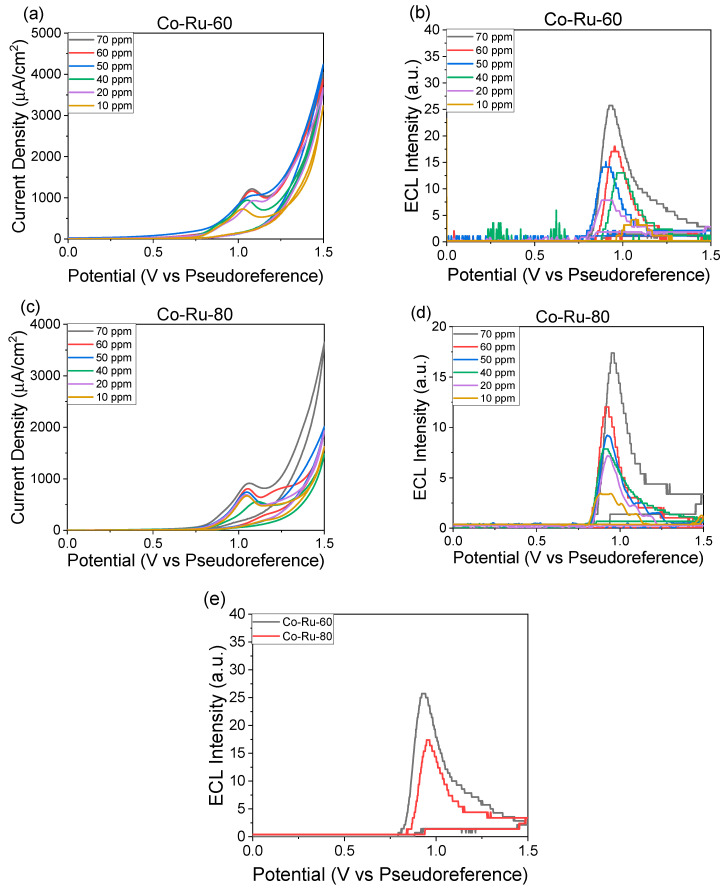
(**a**) Cyclic voltammogram, (**b**) ECL intensity vs. potential response for Gly concentration system for MOF-Co-Ru-60, (**c**,**d**) cyclic voltammogram and ECL intensity response for Gly concentration for MOF-Co-Ru-80, and (**e**) comparative ECL intensity for both systems at 70 ppm.

Gly was detected via ECL on carbon screen-printed electrodes. As shown in Figure 6b, the characteristic electrochemiluminescence plot obtained through cyclic voltammetry for Co-Ru-60 reveals that the oxidation of the ruthenium complex begins at 0.7 V, with a maximum at 1.0 V, Figure 6a, and that of Co-Ru-80 begins at 0.8 V, with a maximum at 1.05 V, Figure 6c; this shift can be attributed to the increase in the ruthenium complex concentration. ECL was obtained with different Gly concentrations between 10 and 70 ppm; for all systems (Figure 6b,d), ECL emissions began at 0.79 V, with a maximum at 0.92 V, and at high concentrations, a better performance was observed with increasing ECL intensities for Co-Ru-60 than for Co-Ru-80 (Figure 6e). This can be attributed to the space in the MOF’s structure that allows more emissions and diffusion between Gly and MOF cavities, which can increase the interaction between ${Ru(bpy)}_{3}^{2+}$ and Gly.

**Figure 7:**
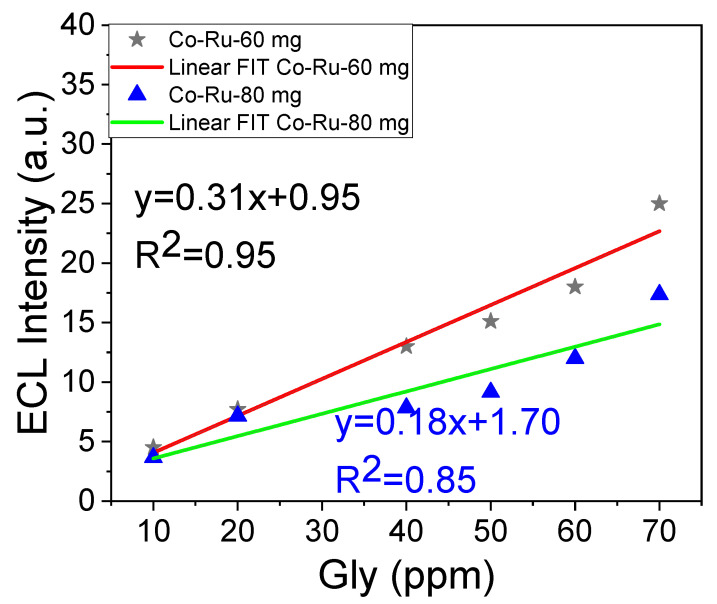
Calibration curve according to Gly concentration for Co-Ru-60 and Co-Ru-80.

As shown in Figure 7, the Gly concentration calibration curve based on the ECL intensity using Co-Ru-60 shows a good correlation and a better intensity at higher Gly concentrations, with an experimental limit of 4 ppm, a theoretical LOD of 20 ppm, and a quantification limit of 70 ppm (LOQ); in contrast, Co-Ru-80 results in a lower ECL intensity than MOF-Co-Ru-60 at high Gly concentrations. The LOD is 7.6 ppm, which is lower than that of Co-Ru-60, and the LOQ is 25 ppm. This corroborates the superior performance of Co-Ru-80, which can be attributed to the morphological difference and ${Ru(bpy)}_{3}^{2+}$ content. Table 2 presents a comparison of the LODs reported from other detection systems. This analysis revealed a good LOD performance compared with other electrochemical sensors previously reported, as well as advantages such as a low cost, ease of assembly, and, above all, the absence of a need for sophisticated equipment such as fluorescence systems. Finally, the stability of the system was realized via pulse differential voltammetry, where the electrochemical process is stable; however, the ECL intensity is not stable, which can be attributed to Gly oxidation, a species that is not reversible for this detection method (Figure S3).

The system does not show electroactive species; the capacitive behavior characterizes the electrochemical response. This behavior was modeled by an equivalent circuit with three elements, R1, Q1, and W1, representing the solution resistance, no ideal capacitance, and a Warburg element associated with mass transport.

**Figure 8:**
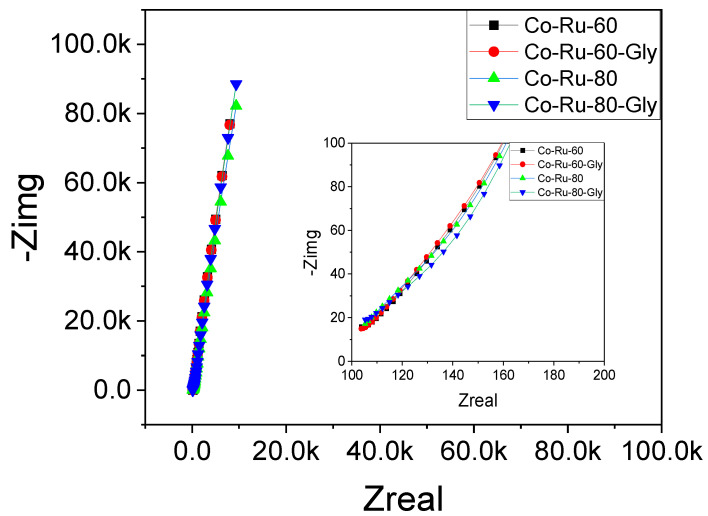
Nyquist plot for Co-Ru-60 and Co-Ru-80.

The results show that the Co-Ru-60 presents the same impedance, and the incorporation of Gly was constantly maintained. For the Co-Ru-80 system, the Gly addition produces an impedance decrease in the order of 20 Ω with respect to the system without Gly. These results are backed up by the values obtained via the Warbug element, which demonstrates a variation after the Gly incorporation, which suggests that mass transport processes are not substantially affected by this analyte (Figure 8).

Table 3 summarizes the parameters obtained by fitting the impedance spectra using the Levenberg–Marquardt algorithm. In this table, one can observe that the α parameter associated with the constant phase is very close to 1 for both systems. This indicates that the CPE can be interpreted as an ideal capacitor. These results are consistent with the ECL response from the sensor that follows a chemical route after Ru oxidation.

Finally, the X^2^ values were obtained from the equivalent circuit adjustment. They reports magnitudes below 0.2, indicating that the model-derived parameters are reliable and adequately represent the electrochemical behavior of the systems studied.

**Figure 9:**
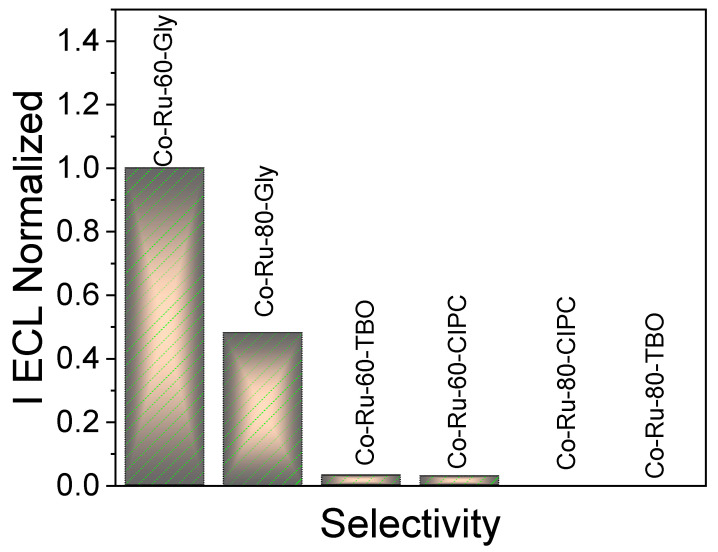
Interference of different pesticides at a concentration of 70 ppm for Co-Ru-60 and Co-Ru-80 systems.

The selectivity analysis was performed using cyclic voltammetry with herbicide models, CIPC and TBO. The performance does not show a response for the Co-Ru-80 systems and shows a lower ECL intensity for the Co-Ru-60 systems (Figure 9); however, this is not representative with an ECL intensity of 0.01, in respect to 1, which can be attributed to the noise because the signal is too slow, see Figures S4 and S5 for the voltammetry cycling and ECL intensity for different herbicides for both systems.

## 4. Conclusions

The synthesis of cobalt–ruthenium–oxalate MOFs was successfully realized with a custom-designed solvothermal reactor that permits magnetic stirring, and the MOF morphology was corroborated by the SEM and structural data from XRD. This study corroborates a change from a monoclinic structure in oxalate to a cubic structure, which suggests that the ${Ru(bpy)}_{3}^{3+}$ complex directly interacts with the MOF components, resulting in a size reduction and luminescence properties in ECL systems. The application of the MOF for glyphosate detection provides a good performance, with an LOD of 7.6 ppm.

## Figures and Tables

**Table 1:** Size of MOFs with different amounts of ${Ru(bpy)}_{3}^{2+}$.

Sample	Size
Co-Ox	5.23 ± 2.0 µm
Co-Ru-30	4.20 ± 1.70 µm
Co-Ru-40	3.22 ± 1.18 µm
Co-Ru-60	2.43 ± 0.61 µm
Co-Ru-80	2.23 ± 0.78 µm

**Table 2:** Comparative LOD for Gly detection.

Detection Technique	Strategy	LOD (ppm)	REF
Square-wave voltammetry (SWL)	n-Si/PANI/HRP	5.4	[28]
MIP@GO/GCE	DPV	1.9	[29]
CoOx nanoparticles	CV/-EIS	0.13	[30]
ZnO NPs	DPV/EIS	0.96	[31]
DNA-AgNCs/Cu^2+^	Fluorescence	0.005	[32]
ECL	Ruthenium: ZnO/AgNp	14	[6]
ECL	Co-Ru-60	20	This work
ECL	Co-Ru-80	7.6	This work

**Table 3:** Adjustment values obtained.

	Unit	Co-Ru-60	Co-Ru-60-Gly	Co-Ru-80	Co-Ru-80-Gly
Chi^2^-Kramers-Kronig	--	0.00151	0.00161	7.322 × 10^−4^	8.853 × 10^−4^
Rsol	Ohms	98.21	99.08	106.6	117.6
CPE	µS^α^/Ω	2.216	2.201	1.255	1.248
alpha	--	0.9723	0.9804	0.9613	0.9642
W	kOhm/sqrt(s)	7.118	8.223	9.343	9.226
chi^2^	--	0.1042	0.1064	0.04614	0.04821

## Data Availability

The original contributions presented in this study are included in the article/Supplementary Material. Further inquiries can be directed to the corresponding authors.

## Supplementary Materials

The following supporting information can be downloaded at https://www.mdpi.com/article/10.3390/bios16030140/s1. Figure S1. morphological analysis and size histogram for MOFs synthesized. Figure S2. SEM-EDS for (a) Co-Ru-60 and (b) Co-Ru-80. Figure S3. stability study of pulse differential voltammetry for Co-Ru-60 and Co-Ru-80, left and right, respectively, for 70 ppm of glyphosate concentration. Figure S4. Selectivity probe by (a) cyclic voltammogram and (b) ECL vs. Potential response (left and right, respectively) for different herbicides with Co-Ru-60 system. Figure S5. Selectivity probe by cyclic voltammogram and ECL vs. Potential response (left and right, respectively) for different herbicides with Co-Ru-80 system.
