# Supplementary material for: The Synthesis of Ru–Co–Oxalate MOFs for an Electrochemiluminescent Glyphosate Sensor
Source: Biosensors (Basel). 2026 Feb 28;16(3):140. doi: 10.3390/bios16030140 (PMC13024222; doi:10.3390/bios16030140)
Supplement: Supplementary file 1 [file biosensors-16-00140-s001.zip › biosensors-4081030-supplementary.pdf]

Supporting information

Communication

The Synthesis of Ru–Co–Oxalate MOFs for an Electrochemiluminescent Glyphosate Sensor

Karina G. Espinosa-Cavazos [1], Joelis Rodríguez-Hernández [1], Carlos Gallardo-Vega [1], Carmen Alvarado-Canché [1], Marco Antonio Castillo [1], Roman Torres-Lubian [1], Perla E. García Casillas [1], Juan Carlos Anaya-Zavaleta [2], Antonio Ledezma-Pérez [1,*] and Arxel de León [3,*]

[1] Centro de Investigación en Química Aplicada, Boulevard Enrique Reyna 140, Saltillo Coahuila 25294, Mexico; karinaespinosa.c@gmail.com (K.G.E.-C.); joelis.rodriguez@ciqa.edu.mx (J.R.-H.); carlos.gallardo@ciqa.edu.mx (C.G.-V.); carmen.alvarado@ciqa.edu.mx (C.A.-C.); marco.castillo@ciqa.edu.mx (M.A.C.); roman.torres@ciqa.edu.mx (R.T.-L.); perla.garcia@ciqa.edu.mx (P.E.G.C.)

[2] Micro and Nanotechnology Research Center, Universidad Veracruzana, Boca del Río 94294, Mexico; juancarlosanayazavaleta@gmail.com

[3] SECIHTI-Centro de Investigación en Química Aplicada, Boulevard Enrique Reyna 140, Saltillo Coahuila 25294, Mexico;

* Correspondence: antonio.ledezma@ciqa.edu.mx (A.L.-P.); arxel.deleon@ciqa.edu.mx (A.d.L.); Tel.: +52-844-4389830 (A.L.-P. & A.d.L.)

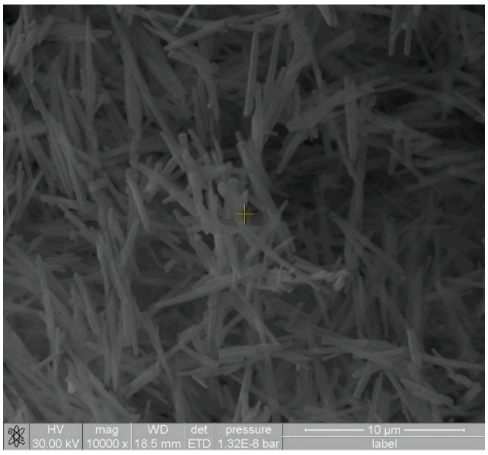

Micrograph Co-Ox

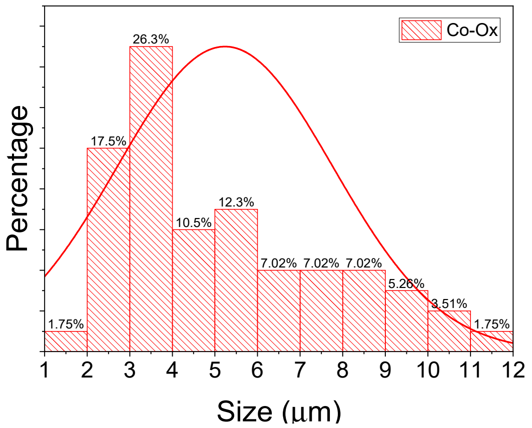

Histogram Co-Ox

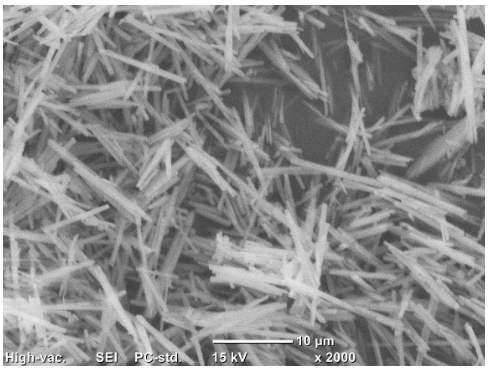

Micrograph Co-Ru-30

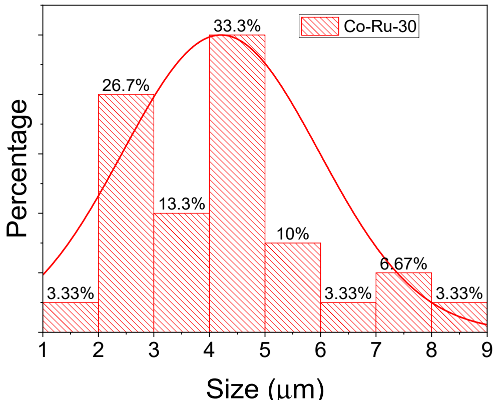

Histogram Co-Ru-30

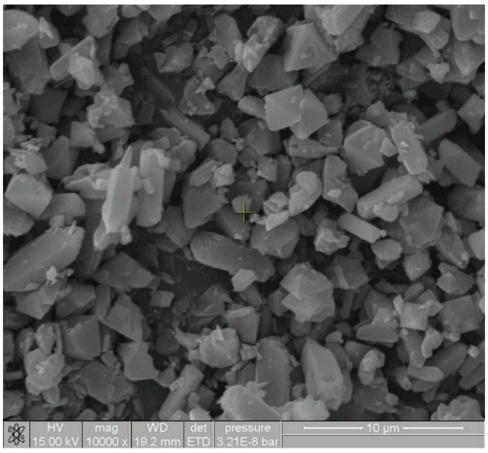

Micrograph Co-Ru-40

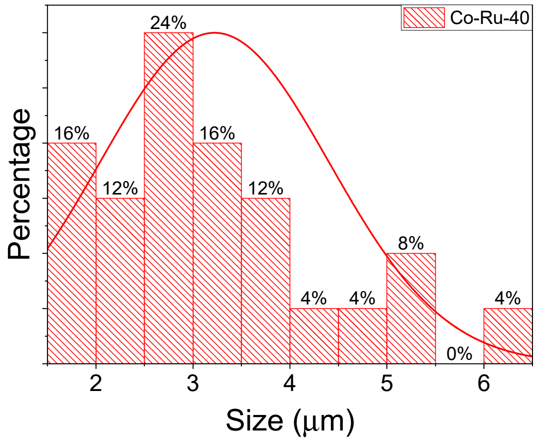

Histogram Co-Ru-40

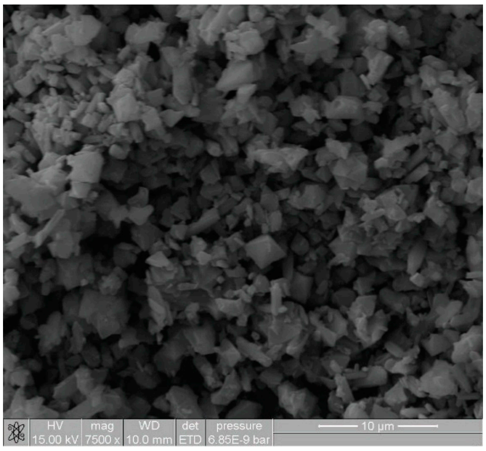

Micrograph Co-Ru-60

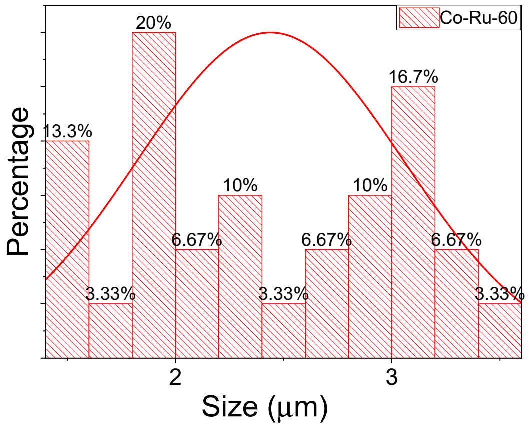

Histogram Co-Ru-60

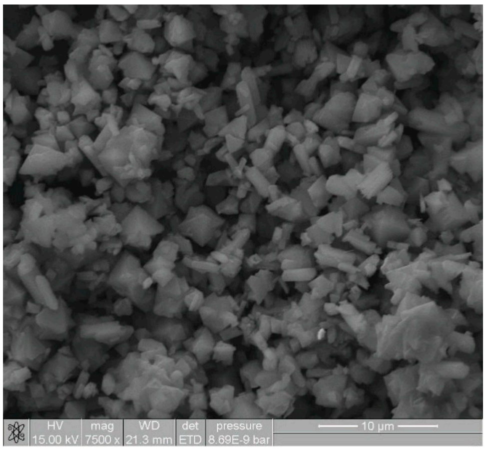

Micrograph Co-Ru-80

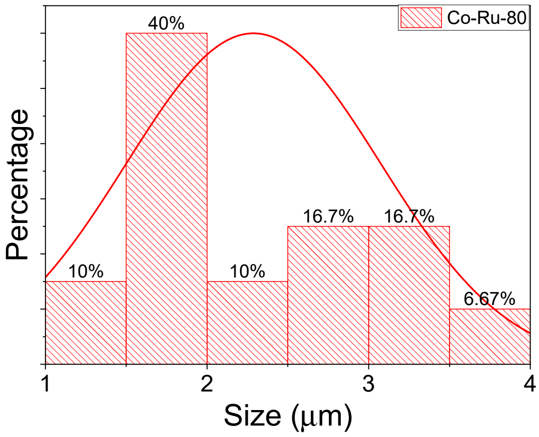

Histogram Co-Ru-80

Figure S1 morphological analysis and size histogram for MOFs synthesized.

a) EDS-SEM Co-Ru-60

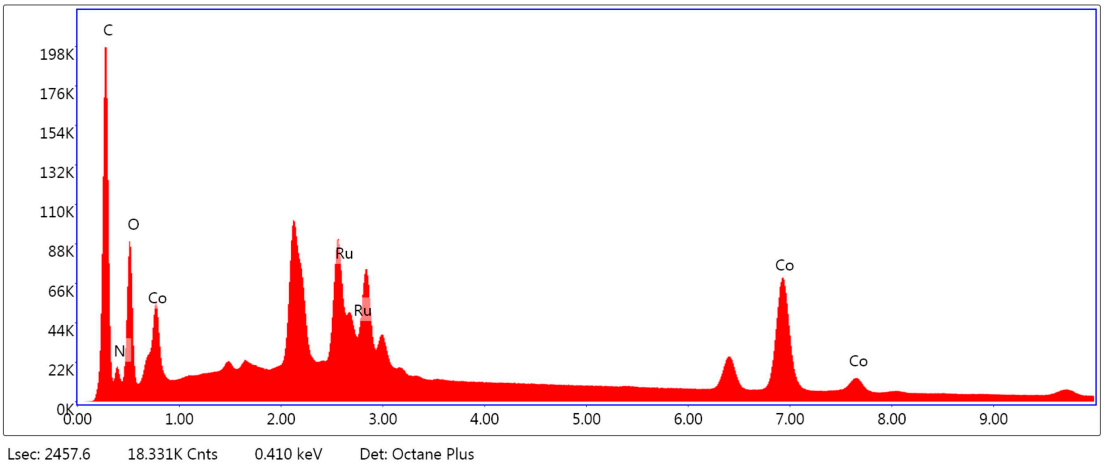

b) EDS-SEM Co-Ru-80

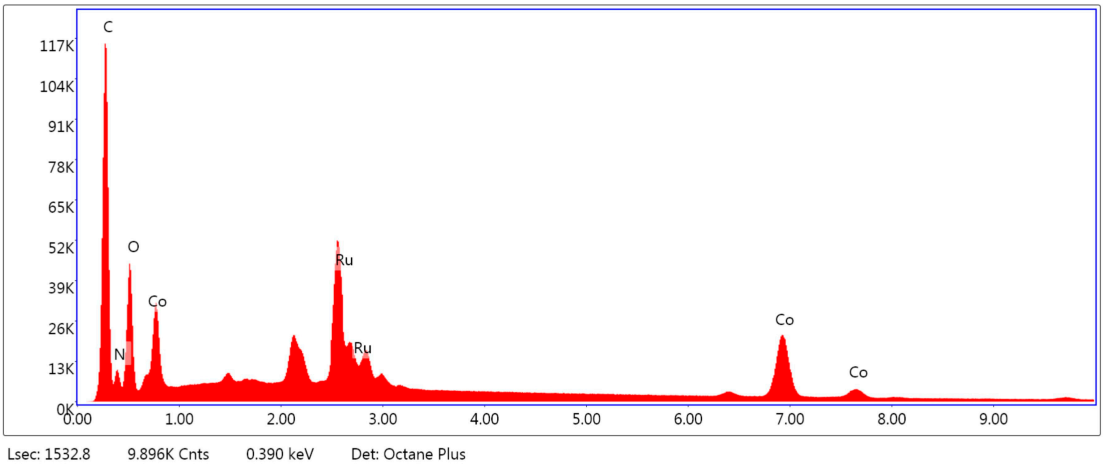

Figure S2. SEM-EDS for a) Co-Ru-60 and b) Co-Ru-80.

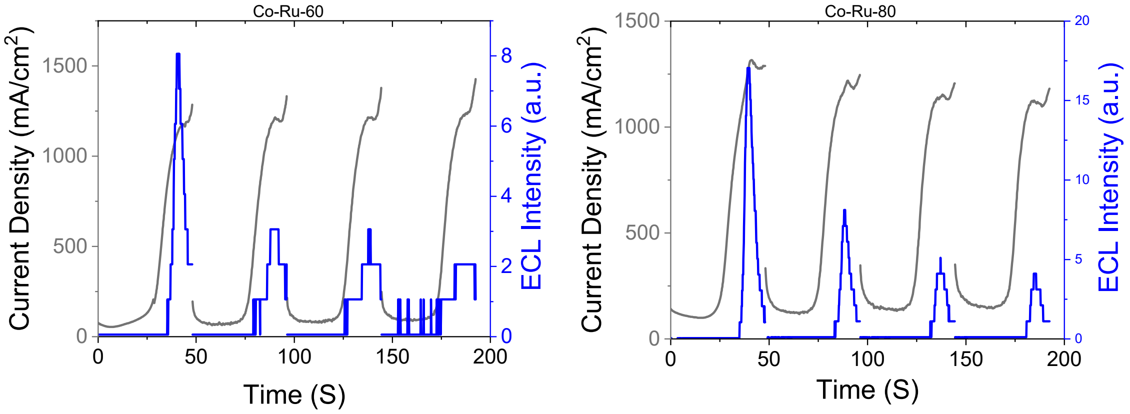

Figure S3. stability study of pulse differential voltammetry for Co-Ru-60 and Co-Ru-80, left and right, respectively, for 70 ppm of glyphosate concentration.

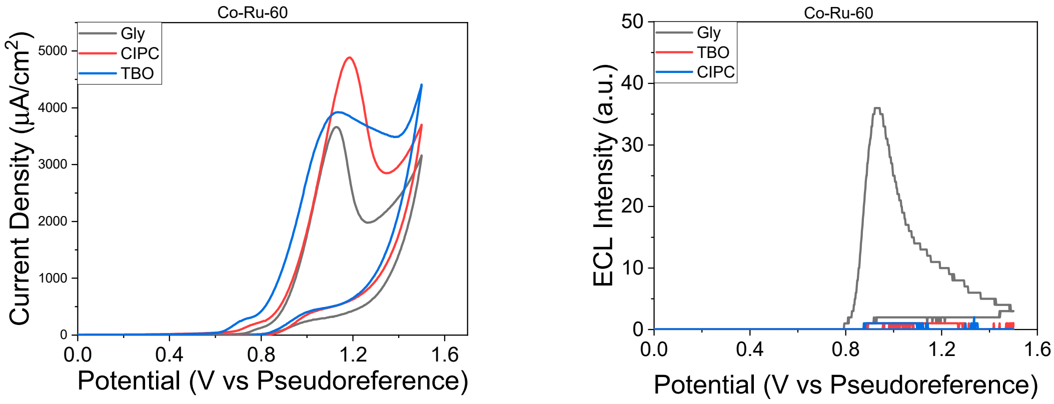

Figure S4. Selectivity probe by a) cyclic voltammogram and b) ECL vs. Potential response (left and right, respectively) for different herbicides with Co-Ru-60 system.

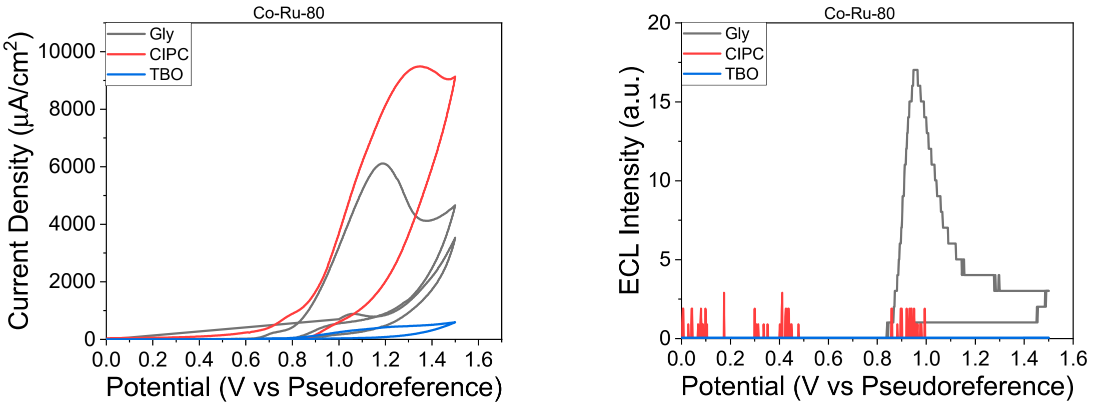

Figure S5. Selectivity probe by cyclic voltammogram and ECL vs. Potential response (left and right, respectively) for different herbicides with Co-Ru-80 system.
